# Whole-tumoral metabolic heterogeneity in ^18^F-FDG PET/CT is a novel prognostic marker for neuroblastoma

**DOI:** 10.1186/s40644-024-00718-3

**Published:** 2024-06-11

**Authors:** Jun Liu, Qinghua Ren, Haonan Xiao, Siqi Li, Lingling Zheng, Xu Yang, Lijuan Feng, Ziang Zhou, Huanmin Wang, Jigang Yang, Wei Wang

**Affiliations:** 1 Department of Nuclear Medicine, Beijing Friendship Hospital, Capital Medical University, 95 Yong An Road, Xi Cheng District, Beijing, 100050 China; 2 Department of Surgical Oncology, Beijing Children’s Hospital, Capital Medical University, National Center for Children’s Health, Beijing, 100045 China; 3 Department of Radiation Oncology and Physics, Shandong Cancer Hospital and Institute, Shandong First Medical University and Shandong Academy of Medical Sciences, No.440, Jiyan Road, 250117 Jinan, Shandong Province China

**Keywords:** Neuroblastoma, Whole-tumoral metabolic heterogeneity, Intra-tumoral metabolic heterogeneity, ^18^F-FDG PET/CT, Prognosis

## Abstract

**Background:**

Neuroblastoma (NB) is a highly heterogeneous tumor, and more than half of newly diagnosed NB are associated with extensive metastases. Accurately characterizing the heterogeneity of whole-body tumor lesions remains clinical challenge. This study aims to quantify whole-tumoral metabolic heterogeneity (WMH) derived from whole-body tumor lesions, and investigate the prognostic value of WMH in NB.

**Methods:**

We retrospectively enrolled 95 newly diagnosed pediatric NB patients in our department. Traditional semi-quantitative PET/CT parameters including the maximum standardized uptake value (SUVmax), the mean standardized uptake value (SUVmean), the peak standardized uptake value (SUVpeak), metabolic tumor volume (MTV) and total lesion glycolysis (TLG) were measured. These PET/CT parameters were expressed as PSUVmax, PSUVmean, PSUVpeak, PMTV, PTLG for primary tumor, WSUVmax, WSUVmean, WSUVpeak, WMTV, WTLG for whole-body tumor lesions. The metabolic heterogeneity was quantified using the areas under the curve of the cumulative SUV-volume histogram index (AUC-CSH index). Intra-tumoral metabolic heterogeneity (IMH) and WMH were extracted from primary tumor and whole-body tumor lesions, respectively. The outcome endpoints were overall survival (OS) and progression-free survival (PFS). Survival analysis was performed utilizing the univariate and multivariate Cox proportional hazards regression. The optimal cut-off values for metabolic parameters were obtained by receiver operating characteristic curve (ROC).

**Results:**

During follow up, 27 (28.4%) patients died, 21 (22.1%) patients relapsed and 47 (49.5%) patients remained progression-free survival, with a median follow-up of 35.0 months. In survival analysis, WMTV and WTLG were independent indicators of PFS, and WMH was an independent risk factor of PFS and OS. However, IMH only showed association with PFS and OS. In addition to metabolic parameters, the International Neuroblastoma Staging System (INSS) was identified as an independent risk factor for PFS, and neuron-specific enolase (NSE) served as an independent predictor of OS.

**Conclusion:**

WMH was an independent risk factor for PFS and OS, suggesting its potential as a novel prognostic marker for newly diagnosed NB patients.

## Background

Neuroblastoma (NB), originating from primitive neural crest cells, is the third common tumor of childhood, accounting for 8–10% of childhood malignancies and 15% of cancer-related deaths in pediatric [1, 2]. Over half of NB patients are high-risk categories with extensive metastatic lesions at initial diagnosis [3]. Common metastatic sites include bone marrow, lymph nodes and liver [4]. Despite advancements in multi-modal treatment such as chemotherapy, radiation therapy, immunotherapy and hematopoietic stem cell support, the prognosis for high-risk NB children with extensive metastases remains poor, with long-term survival rates below 50% [5, 6]. Individualized therapy based on precise tumor staging and risk stratification could improve prognosis. Therefore, accurate assessment of whole-body tumor burden serves as the cornerstone of staging and tailoring individualized treatment for NB patients.

^18^F-FDG PET/CT as a non-invasive imaging examination can provide whole-body tumor metabolic information. Currently, there are many methods to quantify tumor metabolic heterogeneity through ^18^F-FDG PET/CT images including the coefficient of variance (COV) [7], texture analysis [8], fractal analysis [9], heterogeneity factors (HI) [10], cumulative SUV-volume histogram (CSH) [11] and AUC-CSH index [12]. The AUC-CSH index as a novel approach, overcame the limitations of traditional metabolic parameters that could not assess tumor metabolic heterogeneity [13]. It has been widely accepted to assess tumor heterogeneity, which demonstrated a strong correlation with treatment failure and poor prognosis in various tumors such as non-small cell lung cancer, cervical cancer, breast cancer and musculoskeletal tumor [12–16]. Recent studies also calculated the intra-tumoral metabolic heterogeneity (IMH) from the primary tumor, revealing a significant association with event-free survival in NB [17, 18]. However, previous studies indicated that there was significant genetic tumor heterogeneity between primary tumor and metastases [19, 20]. NB also exhibits significant spatial and temporal heterogeneity across various tumor lesions [21]. More than 50% of NB patients have extensive metastases at the time of initial diagnosis [22, 23]. Therefore, evaluating tumor metabolic heterogeneity only based on the primary lesion might underestimate the whole-body tumor heterogeneity of NB patients. To our knowledge, there is a lack of relevant studies in NB to evaluate whole-tumoral metabolic heterogeneity (WMH) based on whole-body tumor lesions. Accurately measuring the WMH is an urgent issue to be addressed.

Meanwhile, most previous studies evaluated traditional PET/CT metabolic parameters derived from the primary lesion [2, 3]. This might underestimate the tumor burden of NB patients with extensive metastases, leading to instable research results [24]. To date, there are no neuroblastoma-related studies that have investigated the prognostic value of PET/CT metabolic parameters extracted from whole-body tumor lesions.

Therefore, our study aims to quantify the WMH, and further investigate the prognostic value of traditional metabolic parameters and WMH in newly diagnosed NB.

## Materials and methods

### Patients

We retrospectively reviewed the nuclear medicine imaging record to identify all pediatric NB patients who underwent clinically indicated ^18^F-FDG PET/CT imaging between January 2018 and December 2019. Patients with baseline ^18^F-FDG PET/CT scan and histopathologic confirmed NB were included. Patients with second tumors or those who had undergone surgery or chemotherapy before ^18^F-FDG PET/CT scan were excluded. Clinical data including gender, age, weight, height, tumor histology characteristics, laboratory test results, treatment details and follow-up information were collected from electronic medical records and phone calls. The study endpoints were progression-free survival (PFS), defined as the time from diagnosis to tumor recurrence, progression or death, and overall survival (OS), determined as the time from diagnosis to death. This retrospective study was approved by the Institutional Review Board of our hospital, and the requirement for written informed consent was waived.

### PET/CT scan parameters

Patients received intravenous administration of ^18^F-FDG (3.7-5.2MBq/kg), after fasting for 4–6 h. All PET/CT scans (Siemens Biograph MCT, Germany) were performed following manufacturer’s recommended clinical protocol approximately 50–70 min after radiopharmaceutical administration. A low-dose CT without contrast medium (tube voltage: 120 keV, tube current: automatic mAs, thickness: 3 mm) were performed from skull to the proximal thigh for localization and attenuation correction before PET scanning. If metastases were suspected in the distal extremities, the scan would be extended from skull to toes including the arms. The whole-body PET scan was performed at 2.5 min per bed position in list-mode model. PET images were reconstructed using the time-of-flight ordered subset expectation maximization algorithm, Gaussian smoothing filter, 2 iterations, 21 subsets, zoom 1.0, pixel size 4.07 mm× 4.07 mm, 3 mm slice thickness and 256 × 256 matrix.

### PET/CT image analysis

We reviewed PET/CT images using an open-source software 3D slicer (version: 4.13.0, https://www.slicer.org). Two experienced nuclear medicine physicians reviewed PET/CT images to identify the primary lesion and metastases. After consensus was reached among two nuclear medicine physicians, tumor lesions were manually outlined in 3D slicer software. The primary tumor was defined as the largest or most dominant tumor lesion. Metastatic lesions included metastatic soft tissue nodes, metastatic lymph nodes and bone/bone marrow involvement. All tumor lesions were manually drawn in the 3D slicer software. Traditional semi-quantitative ^18^F-FDG PET/CT metabolic parameters were measured, including the maximum standardized uptake value (SUVmax), the mean standardized uptake value (SUVmean), the peak standardized uptake value (SUVpeak), metabolic tumor volume (MTV) and total lesion glycolysis (TLG). PSUVmax, PSUVmean, PSUVpeak, PMTV, PTLG were extracted from the primary lesion, and WSUVmax, WSUVmean, WSUVpeak, WMTV, WTLG were extracted from whole-body lesions.

### Intra-tumoral metabolic heterogeneity and whole-tumoral metabolic heterogeneity

**Fig. 1:**
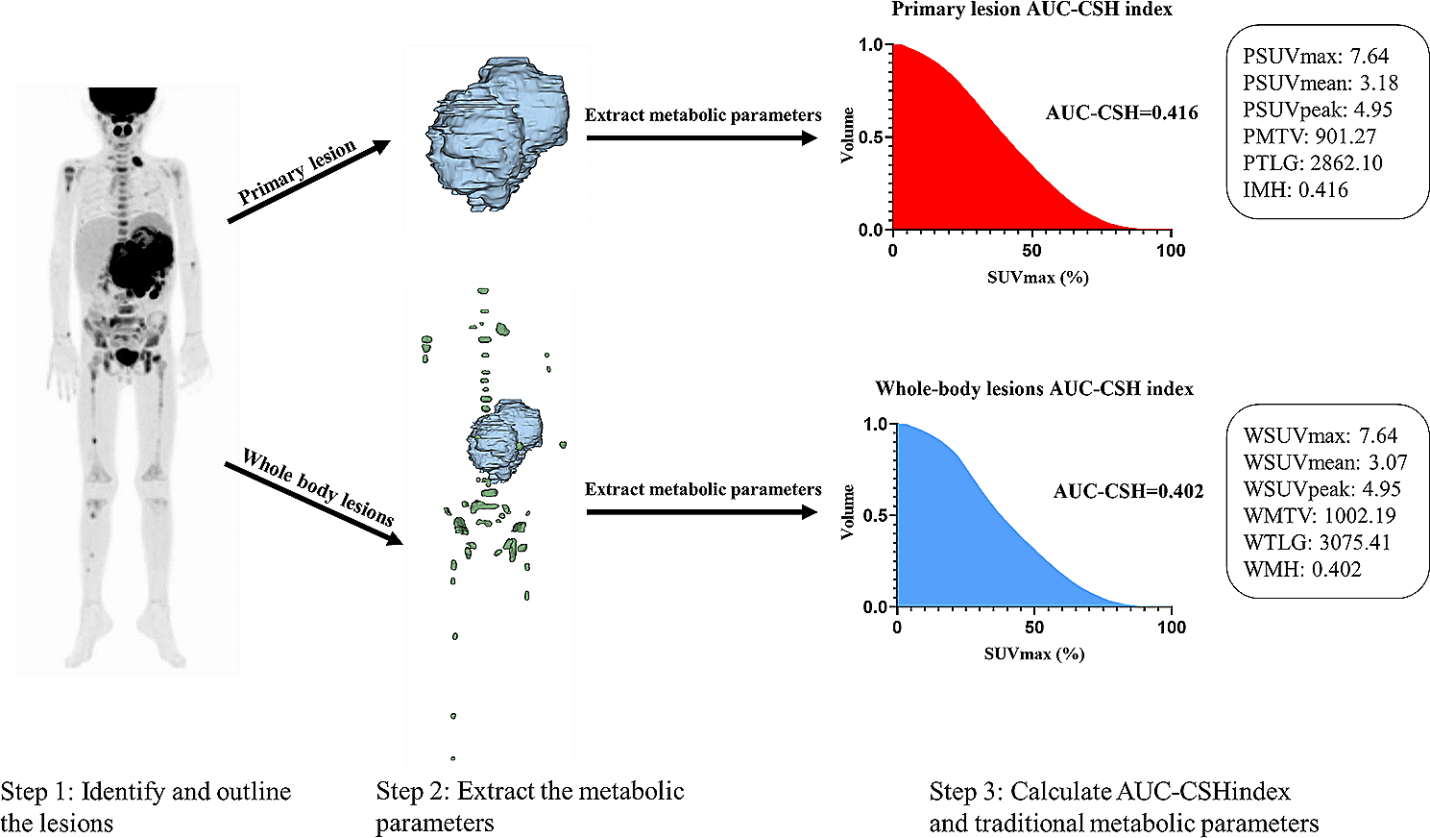
The calculation process of AUC-CSH index and traditional metabolic parameters. Step1: Identify and outline the tumor lesions including primary lesion and metastases. Step2: Extract the tumor metabolic parameters. In this step, we classify the tumor lesions into two forms: primary lesion and whole-body lesions (including primary lesion and metastases). The whole-body lesions as a whole to participate in the subsequent AUC-CSH index. The whole-body lesions would be calculated for only one AUC-CSH index for whole-tumoral metabolic heterogeneity (WMH). Step 3: AUC-CSH index and traditional metabolic parameters based on primary lesion (IMH) and whole-body lesions (WMH) were calculated, separately

IMH was defined as metabolic heterogeneity based solely on the primary lesion. WMH was considered as metabolic heterogeneity derived from whole-body tumor lesions, including both the primary lesion and metastases. IMH and WMH were obtained using the areas under the curve of cumulative SUV-volume histogram index (AUC-CSH index). It was calculated by plotting the percent volume from 0 to SUVmax according to formula: AUC-CSH index=${\int }_{0}^{SUVmax}f\left(x\right)$ [25, 26]. A lower AUC-CSH index reflects heterogeneous metabolic distribution and indicates high tumor metabolic heterogeneity (Detailed calculation procedures are presented in Fig. 1).

### Statistical analysis

Continuous variables were summarized as means ± standard deviations (mean ± SD) or medians with interquartile ranges (IQR), depending on whether they were normal distributions. Categorical variables were presented as counts or percentages. The clinicopathological factors of patients between different groups were compared by using T tests, Mann-Whitney U test, Chi-square test, and Fisher exact tests as appropriate. The correlations between semi-quantitative metabolic parameters were investigated by Spearman correlation tests. Cox proportional hazards regression models were employed for univariate and multivariable survival analysis. All statistical analyses were performed using SPSS (version 26.0) and MedCalc (version 12.7.0). A two-side P-value less than 0.05 was considered statistically significant.

## Results

### Study population

95 newly diagnosed NB patients (male: 46, female: 49) were analyzed in our study. The median age of patients was 2.94 years (interquartile range 1.72–4.73 years). The majority of NB patients (90, 94.7%) had metastases. According to the International Neuroblastoma Staging System (INSS), 1 (1.1%) patient was stage 1, 7 (7.4%) patients were stage 2, 14 (14.7%) patients were stage 3, 73 (76.8%) patients were stage 4. According to Children’s Oncology Group (COG) risk grouping, 6 (6.3%) patients were classified as low-risk, 17 (17.9%) patients as intermediate-risk and 72 (75.8%) patients as high-risk. Ultimately, 90 (94.7%) patients underwent surgery and 93 (97.9%) patients received neoadjuvant chemotherapy. The median follow-up time was 35.0 months (interquartile range 20.5–44.8 months). During clinical follow up, 27 (28.4%) patients died, 21 (22.1%) patients relapsed and 47 (49.5%) patients remained progression-free survival (Table 1).

**Table 1:** Clinical characteristics of patients

Patient Characteristics		Frequency (%)
Age (years)		
	Median	2.94
	Interquartile range	1.72–4.73
Gender		
	Male	46 (48.4%)
	Female	49 (51.6%)
Tumor primary site		
	Abdomen	83 (87.4%)
	Non-Abdomen	12 (12.6%)
Tumor metastasis		
	No	5 (5.3%)
	Yes	90 (94.7%)
INSS		
	Stage 1	1 (1.1%)
	Stage2	7 (7.4%)
	Stage3	14 (14.7%)
	Stage4	73 (76.8%)
COG		
	Low risk	6 (6.3%)
	Medium risk	17 (17.9%)
	High risk	72 (75.8%)
Treatment		
	Neoadjuvant chemotherapy	93 (97.9%)
	Surgery	90 (94.7%)
	Post-operative chemotherapy	93 (97.9%)
Follow up time (months)		
	Median	35.0
	Interquartile range	20.5–44.8
Prognosis		
	Dead	27 (28.4%)
	Relapsed or progression	21 (22.1%)
	Progression-free survival	47 (49.5%)

INSS: International Neuroblastoma Staging System; COG: Children’s Oncology Group;

### Comparing clinicopathological factors and metabolic parameters between the group with and without events

Further analysis was conducted to compare clinicopathological factors and metabolic parameters between the groups with and without events (died or relapsed or progression). There were significant differences in age (*P* = 0.001), INSS (*P* < 0.001), COG (*P* < 0.001), Chromosome 11q (*P* = 0.004), neuron-specific enolase (NSE, *P* < 0.001), serum ferritin (SF, *P* < 0.001), lactate dehydrogenase (LDH, *P* = 0.002), PSUVmax (*P* = 0.030), IMH (*P* < 0.001), WMTV (*P* < 0.001), WTLG (*P* < 0.001), WSUVmax (*P* = 0.001), WSUVpeak (*P* = 0.007) and WMH (*P* < 0.001) between two groups. Tumor primary site (*P* = 0.203), gender (*P* = 0.123), myelocytomatosis viral oncogene neuroblastoma derived homolog (MYCN, *P* = 0.652), Chromosome 1p36 (*P* = 0.696), PMTV (*P* = 0.072), PTLG (*P* = 0.123), PSUVmean (*P* = 0.547), PSUVpeak (*P* = 0.106) and WSUVmean (*P* = 0.760) showed no statistically significance between two groups (Table 2).

**Table 2:** Comparing clinicopathological factors and metabolic parameters between group with and without events

Variable	Without event	With event	χ^2^ / Z	*P*
age(years)			10.687	0.001
<1.5	17 (36.2%)	4 (8.3%)		
≥1.5	30 (63.8%)	44 (91.7%)		
Tumor primary site			1.624	0.203
Abdomen	39 (83.0%)	44 (91.7%)		
Non-Abdomen	8 (17.0%)	4 (8.3%)		
Gender			2.381	0.123
Female	28 (59.6%)	21 (43.8%)		
Male	19 (40.4%)	27 (56.2%)		
INSS			15.686	<0.001
Non-stage 4	19 (40.4%)	3(6.3%)		
Stage 4	28 (59.6%)	45(93.7%)		
COG			17.057	<0.001
Non-high risk	20(42.6%)	3(6.3%)		
High risk	27(57.4%)	45(93.7%)		
MYCN			0.856	0.652
Normal	26 (55.3%)	22 (45.8%)		
Acquire	12 (25.5%)	15 (31.3%)		
Amplification	9 (19.2%)	11 (22.9%)		
Chromosome 1p36			/	0.696
Normal	28 (59.6%)	23 (47.9%)		
Unbalance	8 (17.0%)	11 (22.9%)		
Deletion	10 (21.3%)	12 (25.0%)		
Acquire	1 (2.1%)	2 (4.2%)		
Chromosome 11q			/	0.004
Normal	36 (76.6%)	20 (41.7%)		
Unbalance	3 (6.4%)	6 (12.4%)		
Deletion	7 (14.9%)	20 (41.7%)		
Acquire	1 (2.1%)	2 (4.2%)		
NSE	176.700 (51.850–423.700)	620.000 (199.400-846.250)	-3.930	<0.001
SF	71.300 (34.900-136.300)	236.450 (127.650–441.800)	-4.730	<0.001
LDH	492.000 (331.000-1036.500)	785.500 (582.000-1528.000)	-3.156	0.002
PMTV	124.509 (64.096-325.928)	190.969 (92.312-393.806)	-1.801	0.072
PTLG	311.770 (137.788-727.269)	449.041 (190.966-1135.075)	-1.541	0.123
PSUVmax	4.453 (3.574–5.656)	5.375 (4.300-6.725)	-2.166	0.030
PSUVmean	2.218 (1.741–2.718)	2.392 (1.860–2.764)	-0.603	0.547
PSUVpeak	2.829 (2.240–3.802)	3.498 (2.617–4.466)	-1.615	0.106
IMH	0.505 (0.447–0.555)	0.434 (0.372–0.478)	-3.871	<0.001
WMTV	203.136 (92.436-387.784)	601.844 (445.834-854.694)	-5.471	<0.001
WTLG	394.713 (249.820-930.797)	1447.855 (807.326-2220.685)	-4.920	<0.001
WSUVmax	4.741 (3.891–5.916)	5.971 (5.151–7.038)	-3.208	0.001
WSUVmean	2.358 (1.897–2.911)	2.418 (2.097–2.595)	-0.305	0.760
WSUVpeak	3.113 (2.543–3.909)	3.941 (3.060–4.513)	-2.695	0.007
WMH	0.504 (0.445–0.536)	0.397 (0.352–0.433)	-6.104	<0.001

INSS: International Neuroblastoma Staging System; COG: Children’s Oncology Group; MYCN: myelocytomatosis viral oncogene neuroblastoma derived homolog; NSE: neuron-specific enolase; SF: serum ferritin; LDH: lactate dehydrogenase; SUVmax: the maximum standard uptake value; SUVmean: the mean standardized uptake value; SUVpeak: the peak standardized uptake value; TLG: total lesion glycolysis; WMH: whole-tumoral metabolic heterogeneity; IMH: intra-tumoral metabolic heterogeneity; PSUVmax, PSUVmean, PSUVpeak, PMTV, PTLG were extracted from primary lesion, WSUVmax, WSUVmean, WSUVpeak, WMTV, WTLG were extracted from whole-body lesions

### Comparison of ^18^ F-FDG PET/CT metabolic parameters and tumor metabolic heterogeneity derived from the primary lesion and whole-body lesions

Comprehensive comparisons were performed among different metabolic parameters. In the correlation analysis, PSUVmax and WSUVmax (*r* = 0.901, *P* < 0.001), PSUVmean and WSUVmean (*r* = 0.873, *P* < 0.001), PSUVpeak and WSUVpeak (*r* = 0.870, *P* < 0.001) showed extremely high correlation. Additionally, there was a high correlation between PMTV and WMTV (*r* = 0.725, *P* < 0.001), PTLG and WTLG (*r* = 0.759, *P* < 0.001), IMH and WMH (*r* = 0.772, *P* < 0.001). Our results demonstrated a high positive correlation in metabolic parameters and tumor metabolic heterogeneity between primary lesions and whole-body lesions.

In the differential analysis, no significant difference was observed between PSUVmax and WSUVmax (Z=-1.723, *P* = 0.085), PSUVmean and WSUVmean (Z=-0.784, *P* = 0.433), PSUVpeak and WSUVpeak (*r*=-1.554, *P* = 0.120). However, there were significant differences between PMTV and WMTV (Z=-4.638, *P* < 0.001), PTLG and WTLG (Z=-4.258, *P* < 0.001), IMH and WMH (Z=-2.005, *P* = 0.045). Our study indicated that the tumor metabolic burden (represented by MTV and TLG) and tumor metabolic heterogeneity were significantly different between primary lesions and whole-body lesions (Table 3).

**Table 3:** Comparison of metabolic parameters and tumor metabolic heterogeneity derived from primary lesion and whole-body lesions

Variable	Value	*r*	*P*	Z	*P*
SUVmax		0.901	< 0.001	-1.723	0.085
PSUVmax	5.054 (3.910–6.567)				
WSUVmax	5.623 (4.286–6.746)				
SUVmean		0.873	< 0.001	-0.784	0.433
PSUVmean	2.296 (1.785–2.753)				
WSUVmean	2.374 (1.961–2.683)				
SUVpeak		0.870	< 0.001	-1.554	0.120
PSUVpeak	3.161 (2.467–4.313)				
WSUVpeak	3.604 (2.712–4.458)				
MTV		0.725	< 0.001	-4.638	< 0.001
PMTV	167.754 (76.736-358.598)				
WMTV	390.696 (165.166-677.883)				
TLG		0.759	< 0.001	-4.258	< 0.001
PTLG	359.044 (172.154-896.537)				
WTLG	926.909 (347.252-1750.510)				
Metabolic Heterogeneity		0.772	< 0.001	-2.005	0.045
IMH	0.460 (0.399–0.532)				
WMH	0.434 (0.377–0.504)				

SUVmax: the maximum standard uptake value; SUVmean: the mean standardized uptake value; SUVpeak: the peak standardized uptake value; TLG: total lesion glycolysis; WMH: whole-tumoral metabolic heterogeneity; IMH: intra-tumoral metabolic heterogeneity; PSUVmax, PSUVmean, PSUVpeak, PMTV, PTLG were extracted from primary lesion, WSUVmax, WSUVmean, WSUVpeak, WMTV, WTLG were extracted from whole-body lesions

### Comparing intra-tumoral metabolic heterogeneity and whole-tumoral metabolic heterogeneity in different subgroups

**Fig. 2:**
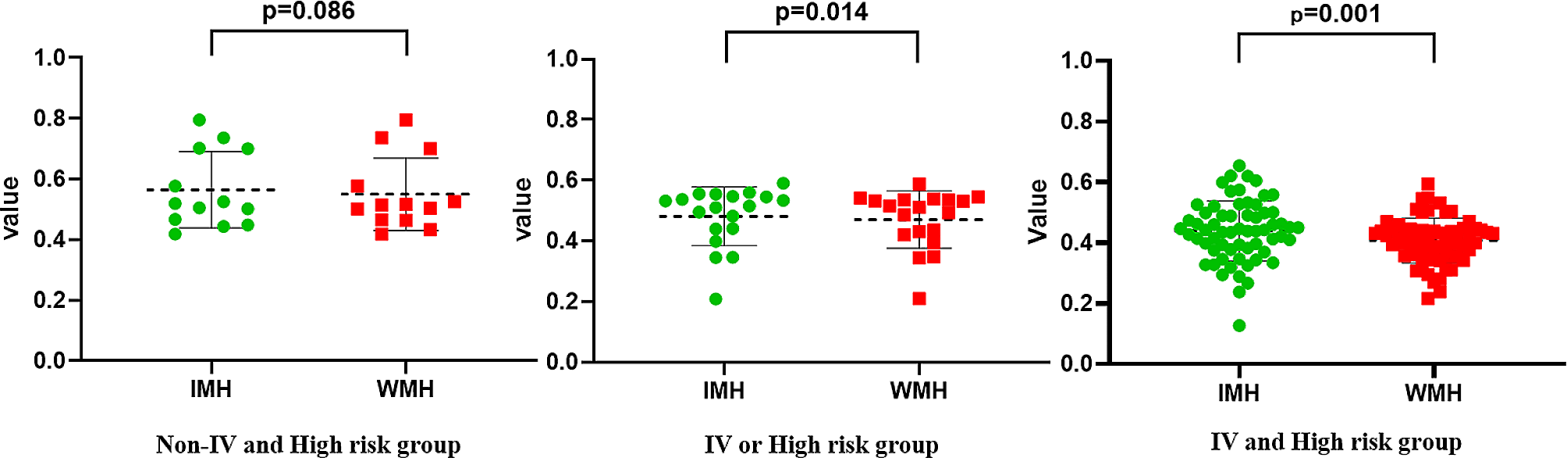
Comparing intra-tumoral metabolic heterogeneity and whole-tumoral metabolic heterogeneity in different subgroups

The differences of IMH and WMH between subgroup of patients were also investigated. NB patients were categorized into three groups according to their INSS staging and COG risk group (non-IV and non-high-risk group, IV or high-risk group, IV and high-risk group). No statistically significant differences were detected in the non-IV and non-high-risk groups (Z=-1.718, *P* = 0.086). While statistically significant differences were observed in IV or high-risk group (Z=-2.461, *P* = 0.014), and IV and high-risk group (Z=-3.389, *P* = 0.001). Our study found that tumor metabolic heterogeneity was significant different in stage IV or high-risk group NB patients (Fig. 2).

### Survival analysis

Clinicopathological factors and metabolic parameters were included in survival analysis. In univariate analysis, age (*P* = 0.017), INSS (*P* = 0.002), COG (*P* = 0.003), Chromosome 11q (*P* = 0.007), NSE (*P* < 0.001), SF (*P* = 0.003), LDH (*P* = 0.008), PSUVmax (*P* = 0.011), PSUVpeak (*P* = 0.035), PMTV (*P* = 0.025), IMH (*P* < 0.001), WSUVmax (*P* = 0.002), WSUVpeak (*P* = 0.006), WMTV (*P* < 0.001), WTLG (*P* = 0.001), and WMH (*P* < 0.001) were significantly associated with PFS. Variables with significant differences in the univariate analysis were included in multivariate analysis. Multivariate analysis identified that INSS (*P* = 0.011), WMTV (*P* < 0.001), WTLG (*P* = 0.004) and WMH (*P* < 0.001) were independent risk factors for PFS (Table 4).

**Table 4:** Survival analysis of prognostic factors for PFS and OS

Variable	PFS Survival analysis				OS Survival analysis			
	Univariate		Multivariate		Univariate		Multivariate	
	HR (95% CI)	*P*	HR (95% CI)	*P*	HR (95% CI)	*P*	HR (95% CI)	*P*
Age	3.483 (1.250–9.702)	0.017			2.121 (0.638–7.046)	0.220		
Gender	1.580 (0.893–2.797)	0.116			1.960 (0.897–4.283)	0.091		
Primary tumor site	0.621 (0.223–1.732)	0.363			0.778 (0.182–3.332)	0.735		
INSS	6.122 (1.898–19.749)	0.002	5.003 (1.444–17.340)	0.011	4.067(0.963–17.179)	0.056		
COG	6.038 (1.873–19.461)	0.003			4.016(0.950-16.964)	0.059		
MYCN	1.143 (0.799–1.634)	0.464			1.709 (1.070–2.729)	0.025		
Chromosome 1p	1.111 (0.823–1.499)	0.491			1.332 (0.900–1.971)	0.151		
Chromosome 11q	1.452 (1.108–1.902)	0.007			1.000 (0.677–1.477)	0.999		
NSE	1.001 (1.001–1.001)	<0.001			1.001 (1.001–1.002)	<0.001	1.001 (1.000–1.001)	0.002
SF	1.001 (1.000–1.002)	0.003			1.001 (1.000–1.002)	0.006		
LDH	1.000 (1.000–1.001)	0.008			1.001 (1.000–1.001)	<0.001		
PSUVmax	1.101 (1.022–1.187)	0.011			1.147 (1.056–1.246)	0.001		
PSUVmean	1.087 (0.840–1.407)	0.525			1.268 (0.955–1.685)	0.101		
PSUVpeak	1.143 (1.010–1.294)	0.035			1.229 (1.072–1.410)	0.003		
PMTV	1.001 (1.000–1.002)	0.025			1.001 (0.999–1.002)	0.301		
PTLG	1.000 (1.000–1.000)	0.111			1.000 (1.000–1.000)	0.275		
IMH	0.010 (0.001–0.123)	<0.001			0.004 (0.000–0.138)	0.003		
WSUVmax	1.108 (1.039–1.182)	0.002			1.145 (1.068–1.227)	<0.001		
WSUVmean	1.080 (0.817–1.428)	0.589			1.293 (0.958–1.746)	0.093		
WSUVpeak	1.166 (1.045–1.301)	0.006			1.233 (1.095–1.389)	0.001		
WMTV	1.002 (1.001–1.002)	<0.001	1.003 (1.002–1.005)	<0.001	1.001 (1.000–1.002)	0.003		
WTLG	1.000 (1.000–1.000)	0.001	0.999 (0.999–1.000)	0.004	1.000 (1.000–1.000)	0.012		
WMH	0.000 (0.000–0.003)	<0.001	0.000 (0.000–0.011)	<0.001	0.000 (0.000–0.001)	<0.001	0.000 (0.000–0.008)	<0.001

INSS: International Neuroblastoma Staging System; COG: Children’s Oncology Group; MYCN: myelocytomatosis viral oncogene neuroblastoma derived homolog; NSE: neuron-specific enolase; SF: serum ferritin; LDH: lactate dehydrogenase; SUVmax: the maximum standard uptake value; SUVmean: the mean standardized uptake value; SUVpeak: the peak standardized uptake value; TLG: total lesion glycolysis; WMH: whole-tumoral metabolic heterogeneity; IMH: intra-tumoral metabolic heterogeneity; PSUVmax, PSUVmean, PSUVpeak, PMTV, PTLG were extracted from primary lesion, WSUVmax, WSUVmean, WSUVpeak, WMTV, WTLG were extracted from whole-body lesions

In univariate analysis of OS, MYCN (*P* = 0.025), NSE (*P* < 0.001), SF (*P* = 0.006), LDH (*P* < 0.001), PSUVmax (*P* = 0.001), PSUVpeak (*P* = 0.003), IMH (*P* = 0.003), WSUVmax (*P* < 0.001), WSUVpeak (*P* = 0.001), WMTV (*P* = 0.003), WTLG (*P* = 0.012), and WMH (*P* < 0.001) were significant associated with OS. However, in multivariate analysis, only NSE (*P* = 0.002) and WMH (*P* < 0.001) remained significant. Therefore, NSE and WMH were identified as independent prognostic risk factors for OS (Table 4).

### Further evaluate tumor metabolic burden and WMH on survival

**Fig. 3:**
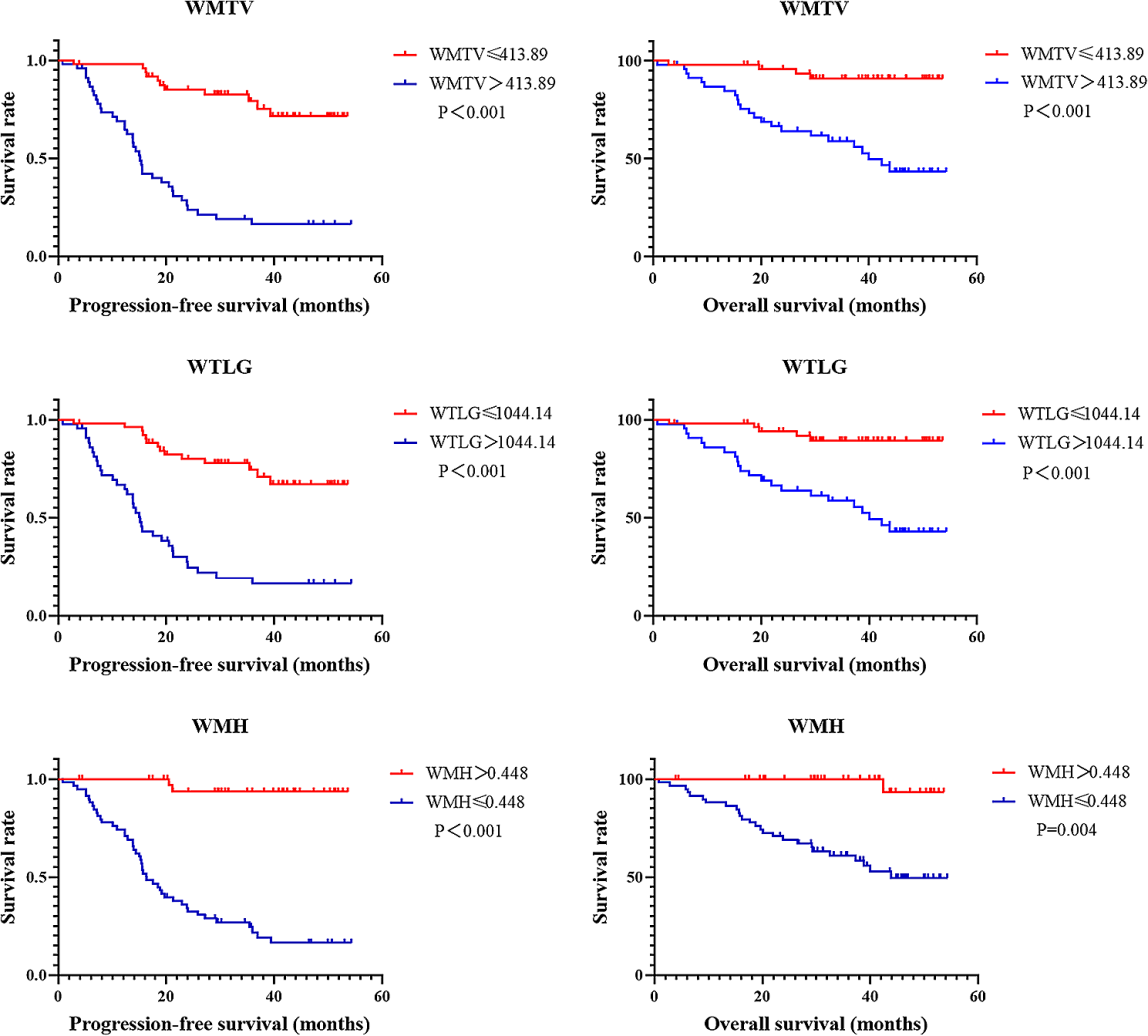
Survival curves based on optimal cut-off value

**Fig. 4:**
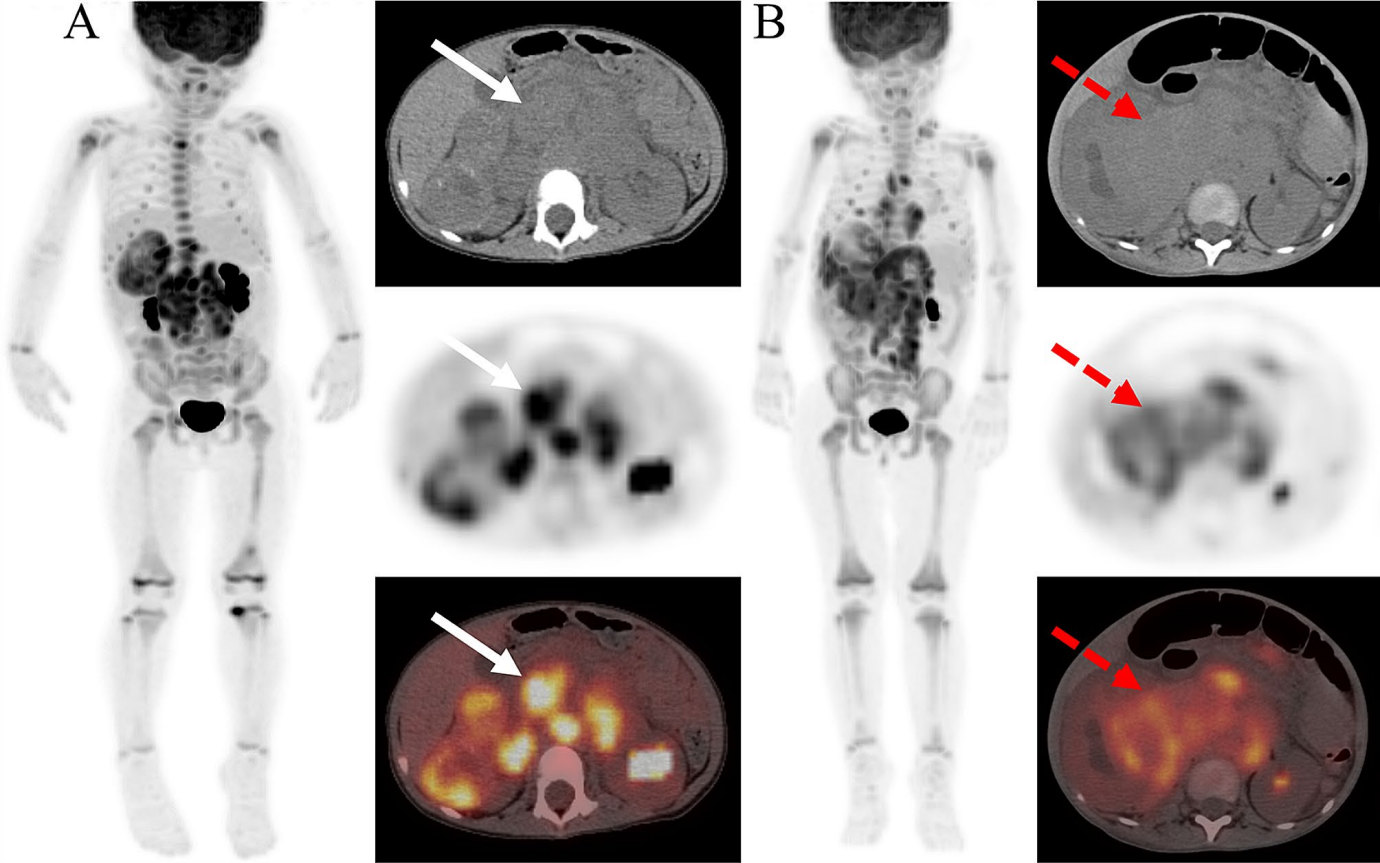
Two representative NB patients with high and low WMH values; A: a 32-month-old girl with a relatively uniform FDG uptake in whole-body tumor (White arrow, IMH:0.443, WMH:0.470), was disease free survival 51.3 months after diagnosis; B: a 29-month-old girl with a heterogeneous FDG uptake in whole-body tumor (Red arrow, IMH:0.463, WMH:0.392), relapsed at 7.1 months, and died at 8.9 months after diagnosis

Based on the results of multivariate survival analysis, we further investigated the predictive effect of whole-body tumor metabolic burden and WHM. The optimal cut-off values for WMTV, WTLG and WMH were 413.14, 1044.14 and 0.448, respectively, determined by the area under the receiver operating characteristic curve. Significant differences were observed in WMTV and WTLG for PFS (*P* < 0.001, *P* < 0.001, respectively) and OS (*P* < 0.001, *P* < 0.001, respectively). Similarly, WMH also manifested significant differences for PFS (*P* < 0.001) and OS (*P* = 0.004) (Fig. 3). Two representative NB patients with high and low WMH values were presented in Fig. 4.

## Discussion

Our study revealed that metabolic parameters based on the primary lesion and whole-body lesions were significantly different. The survival analysis indicated that WMTV and WTLG were independent predictors for PFS, and WMH was an independent predictor for PFS and OS.

NB is one of the most common tumors in children with a highly heterogeneous prognosis [5]. How to accurately assess the prognosis of NB patients has been a pressing clinical challenge. Traditional ^18^F-FDG PET/CT metabolic parameters played an important role in disease diagnosis, recurrence detection, and treatment response evaluation across various tumors [26]. These metabolic parameters have also been widely used for diagnosis, tumor staging, efficacy evaluation and prognostic assessment in NB [27, 28]. Previous study proposed that SUVmax was a vital prognostic factor for OS in NB, while the MTV and TLG were not [2]. In our study, PSUVmax was an important predictor for OS, and PMTV was correlated with PFS, that was slightly different from them. However, some studies also pointed out that MTV and TLG were important predictors for prognosis [3, 28, 29]. There were conflicting opinions regarding the role of traditional metabolic parameters derived from the primary lesion in NB, necessitating further validation and exploration.

One possible reason for these contrasting conclusions was the evaluation of tumor metabolic burden simply based on the primary lesion, potentially underestimating the whole-body tumor burden. In our study, WMTV and WTLG were significantly different from PMTV and PTLG, indicating that the tumor metabolic burden of whole-body lesions was significantly higher than primary lesion. In assessing the metabolic burden of whole-body tumors, Lee et al. only focused on WSUVmax, whereas Ko et al. employed an indirect scoring model to measure WTLG [24, 30]. None of those studies conducted a comprehensive analysis of traditional metabolic parameters. Our study, for the first time, addressed this gap by performing a comprehensive evaluation of traditional metabolic parameters extracted from whole-body tumor lesions in NB. Our study found that WMTV and WTLG were independent predictors for PFS, demonstrating superior prognostic assessment efficacy compared to previous studies [2, 3]. In our study, NSE was also an independent risk factor for OS, demonstrating significant clinical value in assessing the prognosis of NB. However, compare to NSE, PET/CT can provide more information about the tumor in addition to prognostic information. PET/CT could be used for detecting occult lesions, assessing tumor involvement, baseline evaluation of tumors, tumor restaging and so on. NSE primarily serves as an indicative biomarker, whereas PET/CT offers a comprehensive evaluation of the tumor.

Tumors consist of tumor cells and the tumor microenvironment, which undergo interactive evolution and development, resulting in subclonal mutations and tumor heterogeneity [31]. Tumor heterogeneity usually characterized by the difference of tumor metabolism, morphologic, behavioral, angiogenic, proliferative, immunogenic, and metastatic potential [32]. High tumor heterogeneity poses challenges in treatment planning, potentially leading to cancer progression and treatment failure [33]. Recently, there have been increasing interests in evaluating tumor heterogeneity by using ^18^F-FDG PET/CT metabolic parameters [13, 34]. Li et al. used software to extract the tumor metabolic texture features from the primary NB lesion, showed that image heterogeneity texture features were important predictors for PFS [17]. In this study, we use AUC-CSH index to measure tumor metabolic heterogeneity. It is utilized to quantifying the differences of metabolic volume distribution within tumor. Compared to other methods such as COV (standard deviation of SUV/SUVmean) [7] and HI (SUVmax/SUVmean) [10], the AUC-CSH index provides a comprehensive evaluation of metabolic parameters. This index can avoid the influence of extreme values and produce a relative stable outcome. The WMH is derive from the AUC-CSH index. A lower WMH value indicates heterogeneity in tumor metabolism distribution, implying a higher degree of tumor heterogeneity. Additionally, the WMH can overcome the limitation of traditional metabolic parameters, which is unable to evaluate the inhomogeneous uptake within tumors. The WMH demonstrates the feasibility of assessing metabolic heterogeneity of whole-body tumor lesions.

To our knowledge, most studies evaluated tumor metabolic heterogeneity based on the primary tumor lesion [13, 35, 36]. As a highly heterogeneous tumor, NB presents with high spatial and temporal heterogeneity between different tumor lesions [21]. IMH only evaluates the metabolic heterogeneity within primary tumor, and not consider the heterogeneity of metastases. WMH is derived from whole-body tumor lesions, including primary lesion and metastases, which takes into account the metabolic heterogeneity of metastases in NB patients. Compare to IMH, WMH may provide a more accurate depiction of tumor heterogeneity in NB patients with metastases. Our study also found that the tumor metabolic heterogeneity was significantly different between the primary lesion and whole-body lesions. The WMH was significantly lower than IMH in stage IV or high-risk group NB patients. Therefore, accurately evaluating the heterogeneity of NB necessitates the inclusion of whole-body tumor lesions. Our study firstly evaluated the WMH, overcoming the shortcomings of previous studies. Our findings revealed that WMH was an independent risk factor for PFS and OS, offering better prognostic prediction for NB patients than IMH.

Several limitations existed in our study. Firstly, as a small-scale, retrospective and single-center study, our research was subject to selection biases. Secondly, ^18^F-FDG PET/CT was performed in all newly diagnosed NB patients in our study, whereas guidelines generally recommended it for MIBG-negative tumors [37]. Thirdly, different INSS stages and COG risk group NB patients with various treatment regimens were included in our study, which might have impacted on outcomes. Finally, the delineation of lesions, particularly in the determination of primary and metastatic lesions, might partly rely on the subjectivity of operators. Therefore, a large-scale multicenter prospective study should be performed in the future to validate the results of this study.

## Conclusion

This study revealed that WMTV and WTLG were independent predictors for PFS. Furthermore, WMH emerged as an independent risk factor for both PFS and OS, demonstrating its superiority over IMH. These findings suggested that WMH could potentially be a novel prognostic marker for NB.

## Data Availability

No datasets were generated or analysed during the current study.

## Abbreviations

AUC-CSH: areas under the curve of cumulative SUV-volume histogram
COG: Children’s Oncology Group
PFS: Progression-free survival
IMH: Intra-tumoral metabolic heterogeneity
INSS: International Neuroblastoma Staging System
LDH: Lactate dehydrogenase
MTV: Metabolic tumour volume
MYCN: Myelocytomatosis viral oncogene neuroblastoma derived homolog
NSE: Neuron-specific enolase
OS: Overall survival
SF: Serum ferritin
SUVmax: The maximum standard uptake value
SUVmean: The mean standardized uptake value
SUVpeak: The peak standardized uptake value
TLG: Total lesion glycolysis
WMH: Whole-tumoral metabolic heterogeneity
